# Nailfold Capillary Morphology as a Potential Biomarker of Cardiovascular or Metabolic Risk in Children Aged 3–17 Years Prenatally Exposed to Alcohol

**DOI:** 10.1111/acer.70408

**Published:** 2026-09-06

**Authors:** Florencia Anunziata, Julie A. Kable, Marla C. Castañeda, Melanie H. Koslosky, Bretten Pickering, Deanna Beach, Claire D. Coles, Christina D. Chambers, the CIFASD Consortium

**Affiliations:** ^1^ Department of Pediatrics University of California San Diego La Jolla California USA; ^2^ Department of Psychiatry and Behavioral Sciences Emory University School of Medicine Atlanta Georgia USA; ^3^ Department of Pediatrics Emory University School of Medicine Atlanta Georgia USA

## Abstract

**Background:**

Emerging evidence indicates that individuals with prenatal alcohol exposure (PAE) may be at risk for cardiometabolic dysfunction. Nailfold capillaroscopy (NFC) is a noninvasive method for visualizing microvascular dysfunction, which often precedes clinical disease. In this study, we examined whether children and adolescents with confirmed PAE differed in nailfold capillary morphology from unexposed peers.

**Methods:**

We used a cross‐sectional design to compare nailfold capillary morphology in children and adolescents aged 3–17 years with confirmed PAE versus no or minimal PAE, enrolled as part of a multi‐site Collaborative Initiative on Fetal Alcohol Spectrum Disorders (CIFASD) study. Participants completed nailfold capillaroscopy using a high‐resolution video microscope (Optilia Instruments). Quantitative measures included capillary density, average capillary width (μm), and average inter‐capillary distance (μm). Morphological abnormalities (enlarged, branched, broken, crossed, and multi‐crossed capillaries) were also assessed. Separate multivariable linear regression models were fit for each outcome, adjusting for child age and sex.

**Results:**

The sample included 27 children with PAE (mean age = 8.3 ± 4.0 years; 52% male; 70% White; 66% Hispanic) and 10 with no/minimal PAE (mean age = 11.2 ± 4.7 years; 50% male; 70% White; 60% non‐Hispanic). After adjusting for age and sex, participants with PAE showed lower capillary density (mean = 9.4 ± 2.6 vs. 13.2 ± 3.0; *p* < 0.001), greater average intercapillary distance (mean = 171.3 μm ± 28.9 vs. 136.6 μm ± 27.3; *p* < 0.01), greater average capillary width (mean = 43.9 μm ± 11.4 vs. 35.2 μm ± 6.1; *p* < 0.01), and a higher percentage of enlarged capillaries (mean = 71.4 ± 19.1 vs. 46.6 ± 13.8; *p* < 0.001) compared to controls. Age was negatively associated with density (*p* < 0.05), whereas the child's sex was associated with width (*p* < 0.01), with females exhibiting wider capillaries.

**Conclusion:**

These findings, to our knowledge, provide the first characterization of nailfold microvascular morphology in children and adolescents with PAE and support NFC as a potential early biomarker of cardiometabolic risk.

## Graphical abstract

Children with prenatal alcohol exposure (PAE) exhibit early alterations in their microvasculature. Using nailfold capillaroscopy, a noninvasive imaging technique, we compared nailfold capillary morphology in 27 children with PAE and 10 unexposed peers (3–17 years). Children with PAE had lower capillary density, greater intercapillary distance, and wider and more enlarged capillaries than unexposed peers, independent of age and sex. These early microvascular changes may serve as a noninvasive biomarker of future cardiometabolic risk in this population.

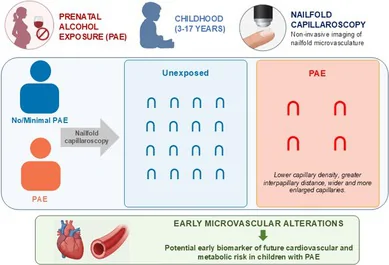

## 1 Introduction

Prenatal alcohol exposure (PAE) is the leading preventable cause of congenital anomalies and neurodevelopmental disabilities worldwide, with the estimated prevalence of fetal alcohol spectrum disorders (FASD) ranging from 1% to 5% among school‐age children in the United States (Lange et al. 2017; May et al. 2018). While the neurobehavioral and cognitive consequences of PAE have been characterized, the long‐term cardiometabolic risks in exposed individuals remain poorly understood (Moore and Riley 2015; Weeks et al. 2020, 2026).

Emerging evidence from both clinical and preclinical studies suggests that PAE contributes to long‐term cardiovascular and metabolic risk, consistent with the developmental origins of health and disease (DOHaD) framework (Barker 2003; Gluckman et al. 2007; Hanson and Gluckman 2008). In a cohort of adults with FASD, PAE has been associated with increased risk of Type 2 diabetes mellitus, low high‐density lipoprotein (HDL) cholesterol, and elevated triglycerides relative to matched controls, with 46.9% of FASD patients exhibiting two or more metabolic abnormalities compared with 26.2% of controls (Weeks et al. 2020). In pediatric populations, PAE has been linked to elevated blood pressure, independent of race/ethnicity and obesity status (Cook et al. 2019). More recent evidence suggests early autonomic and cardiorenal dysregulation, including elevated resting heart rate and altered renal sodium handling in children with FASD (Reid et al. 2025), as well as increased diabetes risk, arterial stiffness, and vascular dysfunction in adults with PAE (Kable et al. 2021).

Findings from preclinical models further support these observations. For example, studies in zebrafish have shown that embryonic alcohol exposure may predispose offspring to later metabolic dysfunction, including visceral adiposity, elevated body mass index (BMI), and fasting hyperglycemia in adulthood, particularly among males (Weeks et al. 2020). Together, these findings suggest that PAE may act as an early‐life contributor to later cardiometabolic risk, with vascular alterations potentially emerging during childhood and contributing to adverse health outcomes across the lifespan. However, reliable, noninvasive tools to detect early subclinical vascular compromise in pediatric populations impacted by PAE remain limited.

Microvascular dysfunction is an early, subclinical indicator of cardiovascular, metabolic, inflammatory, and immune risk that often precedes overt clinical disease by years to decades (Ingegnoli et al. 2005; Ingegnoli and Herrick 2013; Komai et al. 2024; Maranhão et al. 2016; Melsens et al. 2023; Smith et al. 2020). Nailfold capillaroscopy (NFC) is a validated, highly sensitive, cost‐effective, simple, safe, and noninvasive method that provides direct, in vivo visualization of peripheral microvascular morphology (Andrés et al. 2024; Cutolo et al. 2000; Gorasiya et al. 2022; Maranhão et al. 2016; Nakajima et al. 2022; Tavakol et al. 2015). NFC has been applied across diverse clinical contexts for more than 50 years (Komai et al. 2024; Smith et al. 2020, 2023). Key parameters captured by NFC, including capillary density and the prevalence of crossed, branched, and enlarged capillaries, have been associated with systemic vascular health, endothelial function, and cardiometabolic risk, primarily in adult populations, with more limited investigation in pediatric cohorts (Emrani et al. 2017; Komai et al. 2024; Nakajima et al. 2022; Tavakol et al. 2015). Reduced capillary density has been linked to impaired tissue perfusion and microvascular rarefaction in Type 2 diabetes, hypertension, and obesity (Maldonado et al. 2017; Maranhão et al. 2016; Okabe et al. 2024), while enlarged capillaries are a feature of inflammatory and autoimmune conditions in both adults and children (Cutolo et al. 2000; Ingegnoli et al. 2005; Schonenberg‐Meinema et al. 2021; Smith et al. 2020; Tavakol et al. 2015). Furthermore, studies applying NFC in healthy cohorts have further demonstrated that lifestyle and metabolic factors, including body mass index (BMI), sleep, physical activity, psychological stress, and alcohol intake, influence capillary morphology in measurable ways (Gorasiya et al. 2022; Nakajima et al. 2022).

In pediatric populations, NFC has been primarily applied in the context of rheumatologic conditions, such as juvenile dermatomyositis and systemic lupus erythematosus (Gokcen 2025; Herrick et al. 2021; Ingegnoli and Herrick 2013; Smith et al. 2023). Importantly, normative data for children are limited, and standardized pediatric reference values have not been established (Bergkamp et al. 2023; Melsens et al. 2023). Despite this, it has been shown that capillary density increases with age from infancy through approximately the first decade of life, while younger children tend to have wider capillary loops than older children and adults (Gokcen 2025).

Despite the expanding clinical applications of NFC, the microvasculature of individuals with PAE has not been examined. Given the increased predisposition of individuals with PAE to later cardiometabolic disease, and the sensitivity of NFC to early subclinical vascular alterations, NFC represents a compelling tool for characterizing microvascular health in this at‐risk population. The present study aimed to fill this gap by characterizing nailfold capillary morphology in a pediatric sample with confirmed PAE compared to unexposed peers, with the broader goal of identifying early, noninvasive indicators that could support timely preventive screening and strategies for individuals affected by PAE.

## 2 Materials and Methods

### 2.1 Study Design and Setting

The main cross‐sectional study was designed to examine clinical and subclinical indicators of current or future chronic disease among children and adolescents aged 3–17 years with and without confirmed PAE. The study is conducted as part of the Collaborative Initiative on Fetal Alcohol Spectrum Disorders (CIFASD), a multisite consortium funded by the National Institute on Alcohol Abuse and Alcoholism (NIAAA), across two recruitment sites: the University of California San Diego (UCSD) and Emory University. The main study protocol included a comprehensive single‐visit clinical assessment consisting of physical examinations by a study pediatrician (anthropometric measurements, blood pressure, hearing, and vision testing), parent‐completed health questionnaires (dietary habits, physical activity, sleep, anxiety, depression, and adverse childhood experiences), biomarker sampling from blood and saliva (glucose, lipids, hemoglobin A1C, C‐reactive protein, thyroid levels, inflammatory cytokines, miRNAs, and telomere length), cognitive assessments using the NIH Toolbox, 3D facial imaging for FASD‐related dysmorphology, and nailfold video capillaroscopy examinations. The present report focuses on the capillaroscopy component and presents preliminary data from a subset recruited only from the UCSD site.

All study procedures were approved by the Institutional Review Boards at UCSD and Emory University. Written informed consent was obtained from parents or legal guardians; age‐appropriate assent was obtained from participants 7 years of age and older.

### 2.2 Participants

The full study targets a sample of approximately 200 participants (120 with confirmed PAE and 80 no/minimal PAE) recruited across both sites. Children with PAE are being recruited through referrals from clinical providers, community partners, and existing CIFASD registries. Participants from the no PAE group are being recruited through community‐based channels, social media, and enrolled in prior studies. Preliminary data presented here include *N* = 37 participants evaluated at UCSD: 27 with confirmed PAE and 10 with no or minimal PAE.

Inclusion criteria for the PAE group required documented or confirmed PAE, defined as maternal self‐report of more than minimal alcohol use during pregnancy or a clinical diagnosis within FASD, including fetal alcohol syndrome (FAS), partial FAS (pFAS), alcohol‐related neurodevelopmental disorder (ARND), or alcohol‐related birth defects (ARBD) as defined by diagnostic guidelines (Hoyme et al. 2016). Individuals with documented PAE may not have completed a comprehensive diagnostic evaluation at the time of this study; therefore, the absence of a formal diagnosis should not be interpreted as the absence of FASD. Among PAE participants, diagnoses included FAS (*n* = 1, 3.7%), pFAS (*n* = 4, 14.8%), ARND (*n* = 7, 25.9%), and PAE without a formal FASD diagnosis (*n* = 15, 55.5%); all diagnoses were established by expert dysmorphologists. Participants in the no PAE group were required to have no or minimal PAE, defined as maternal report of less than two confirmed drinking occasions during pregnancy with no binge episodes (i.e., 4 or more standard drinks per occasion; Gosdin et al. 2022). Exclusion criteria for both groups included inability to complete the assessment protocol.

### 2.3 Nailfold Capillaroscopy Procedures

Nailfold capillaroscopy was performed using a noninvasive high‐resolution video microscope (Optilia FlexZoom, Optilia Instruments, Solna, Sweden), with a larger magnification lens coupled with a digital video camera and laptop for data encoding and analysis, which provided magnified visualization of the peripheral microcirculation at the nailfold of the fingers (study outcomes). Prior to image capture, children were asked to remove nail polish and wash their hands; one to two drops of cedar oil were applied to the nailfold to reduce light reflection and enhance visualization of the capillary structure through the transparent epidermis.

All capillaroscopic assessments were performed by a single trained examiner. Images were captured at the nailfold of the second, third, or fourth digit (pointer, middle or ring fingers) of either hand (Kayser et al. 2019; Smith et al. 2020). For each participant, a minimum of three nonoverlapping high‐quality fields of view were obtained and recorded for subsequent offline analysis. Images were excluded from analysis if they were of insufficient quality due to excessive motion artifact, poor nail transparency, or skin conditions obscuring capillary visualization.

Nailfold capillary images were quantitatively and qualitatively analyzed. Quantitative parameters extracted from each image included: (1) *capillary density*, defined as the number of capillary loops visible per millimeter of nailfold (capillaries/mm; (Emrani et al. 2017)); (2) *inter‐capillary distance*, defined as the mean distance in micrometers (μm) between adjacent capillary loops; and (3) *capillary width*, defined as the mean diameter in micrometers (μm) of capillary loops measured at the apical portion of the loop.

Morphological qualitative characterization was performed according to established classification schemes (Ingegnoli et al. 2005; Ingegnoli and Herrick 2013; Melsens et al. 2023; Smith et al. 2020, 2023). Capillaries were classified as morphologically *normal* (hairpin or U‐shaped configuration, without crossing), *crossing* or *multi‐crossing* (loops crossing once or more), *branched* (bifurcated loops), *broken* (one side of the capillary is discontinued), or *enlarged* (visible capillary thickening during image review) (Komai et al. 2024; Smith et al. 2020, 2023). Proportions of enlarged, broken, branched, crossed, and multi‐crossed capillaries within each participant's total counted capillaries were computed as a continuous variable and served as a primary morphological outcome. Microcapillaries were imaged using a standard 200× magnification lens with cool white LED illumination and a 5‐megapixel camera (2000 × 1500‐pixel resolution). Images were then analyzed by a trained reader, using the Optilia image analysis software (Optipix). Inter‐rater agreement between two independent raters (UCSD and Emory University) across all capillary metrics was high (*r* > 0.80).

Demographic information was collected at the enrollment visit via structured parent interview. Variables assessed included child age, sex assigned at birth, and race/ethnicity, classified according to NIH reporting standards.

### 2.4 Statistical Analysis

Descriptive statistics were computed for all demographic and capillaroscopic variables by exposure group (PAE vs. Unexposed). All continuous variables were tested for normality. Variables approximately normally distributed were summarized as means ± standard deviations (SD), and those with evidence of nonnormal distributions, particularly those with right skew or zero‐inflation (crossed and multicrossed capillaries), were summarized as medians with interquartile ranges (IQR). Categorical variables were reported as frequencies and percentages. Group comparisons of demographic and nailfold capillary characteristics were performed using independent‐samples Student's *t*‐tests for normally distributed continuous variables, Mann–Whitney *U* tests for nonnormally distributed continuous variables, and Pearson's chi‐squared (*χ* ^2^) tests for categorical variables.

Multivariable linear regression analyses were subsequently conducted to assess whether PAE group differences in capillary parameters remained significant after adjustment for child age and sex—covariates identified a priori based on prior literature demonstrating their influence on nailfold capillary morphology (Emrani et al. 2017; Nakajima et al. 2022; Tavakol et al. 2015). Regression coefficients (*β*) and 95% confidence intervals (CI) were reported. Model assumptions, including residual distributions and influential observations, were evaluated for all regression models. Sensitivity analyses were conducted when appropriate.

Morphological outcomes with nonnormal distributions (percent crossed and multi‐crossed capillaries) were reported descriptively only; adjusted models did not demonstrate significant associations. Two‐sided tests were used throughout; statistical significance was defined as *p* < 0.05. All analyses were performed using SAS (version 9.4; SAS Institute, Cary, NC).

## 3 Results

### 3.1 Sample Characteristics

Participant sociodemographic characteristics by PAE group (*N* = 37) are described in Table 1. Participants with PAE (*n* = 27) were younger on average than the unexposed group (*n* = 10), although this difference was not statistically significant. The groups were similar with respect to sex distribution and racial composition, with most participants in both groups identified as White. Participants with PAE were more likely to identify as Hispanic, whereas those without PAE were more likely to identify as non‐Hispanic. No statistically significant differences in other demographic characteristics were observed between groups.

**TABLE 1:** Participant sociodemographic characteristics by prenatal alcohol exposure group (PAE) (*n* = 37).

	No PAE (*n* = 10)	PAE (*n* = 27)	*p* value
	**Mean (SD)**	**Mean (SD)**	
Age (years)	11.2 (4.7)	8.3 (4.0)	0.0680
Sex	***n* (%)**	***n* (%)**	0.9203
Female	5 (50)	13 (48.1)	
Male	5 (50)	14 (51.9)	
Race			0.5933
White	7 (70)	19 (70.4)	
Asian	0 (0)	2 (7.4)	
Native American	2 (20)	3 (11.1)	
More than one/other	1 (10)	3 (11.1)	
Ethnicity			0.1423
Hispanic	4 (40)	18 (66.6)	
Non‐Hispanic	6 (60)	9 (33.3)	

Abbreviation: SD, standard deviation.

### 3.2 Nailfold Capillary Characteristics

Group comparisons of nailfold microvascular characteristics by PAE group are shown in Table 2. Briefly, participants with PAE showed lower mean capillary density, greater inter‐capillary distance, wider capillary loops, and a higher percentage of enlarged capillaries compared to those in the unexposed group. Higher proportions of broken and branched capillaries were also observed among children with PAE, although this difference was not statistically significant. Crossed and multi‐crossed capillaries were infrequent in both groups and were presented using medians and interquartile ranges because of nonnormal distributions.

**TABLE 2:** Nailfold microvascular characteristics by prenatal alcohol exposure group (PAE) (*n* = 37).

	No PAE (*n* = 10)	PAE (*n* = 27)	*p* value
	**Mean (SD)**	**Mean (SD)**	
Capillary density (capillaries/mm)	13.2 (3.0)	9.4 (2.6)	< 0.001
Average intercapillary distance (μm)	136.6 (27.3)	171.3 (28.9)	< 0.01
Average capillary width (μm)	35.2 (6.1)	43.9 (11.4)	< 0.01
% Enlarged capillaries	46.6 (13.8)	71.4 (19.1)	< 0.001
% Broken capillaries	26.0 (15.5)	37.4 (21.8)	0.1408
% Branched capillaries	20.8 (17.1)	27.5 (18.9)	0.3376
	**Median (IQR)**	**Median (IQR)**	
% Crossed capillaries	26.0 (0.0–33.3)	18.0 (11.1–38.5)	0.5211
% Multi‐crossed capillaries	0.0 (0.0–0.0)	0.0 (0.0–0.0)	0.5559

Abbreviations: IQR, interquartile range; SD, standard deviation.

### 3.3 Adjusted Regression Analyses

**FIGURE 1:**
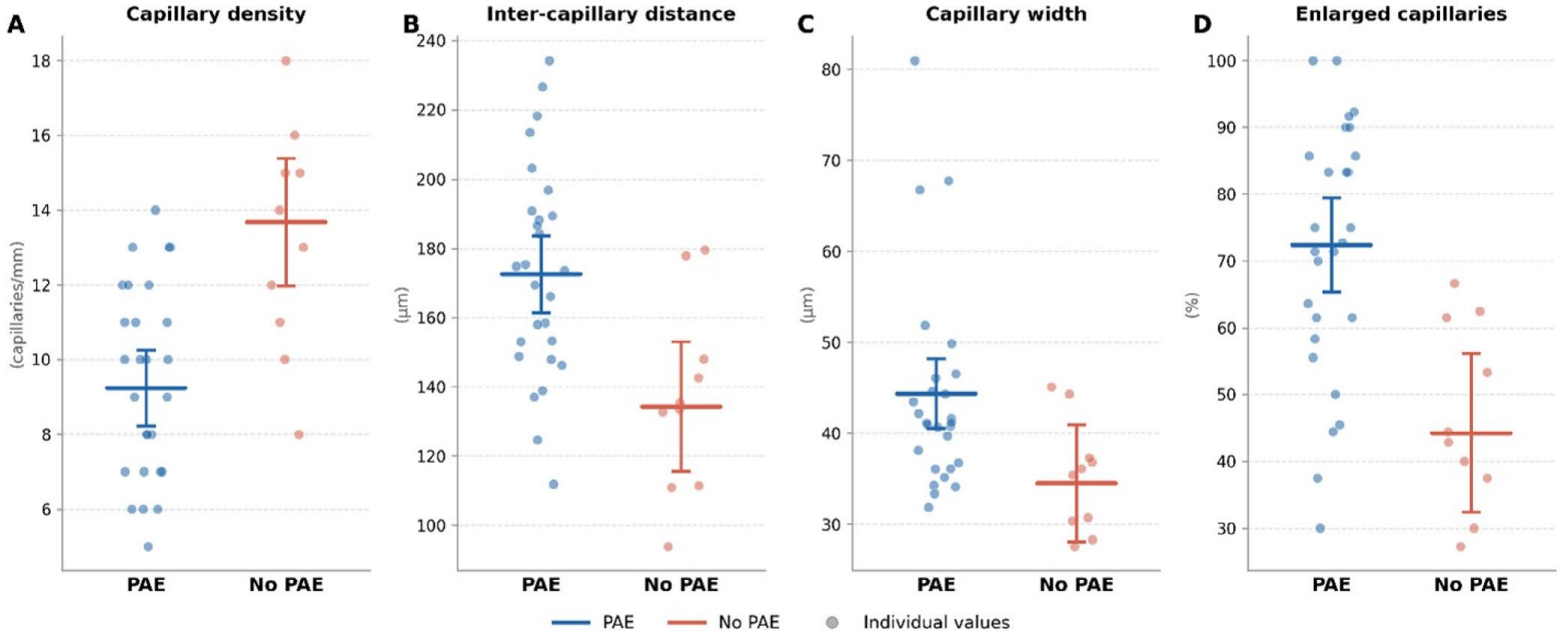
Nailfold microvascular characteristics by prenatal alcohol exposure (PAE) status. Adjusted least‐square means (LS means) and 95% confidence intervals (CIs) from linear regression models adjusted for age and sex are shown for (A) capillary density, (B) intercapillary distance, (C) capillary width, and (D) percentage of enlarged capillaries. Dots represent individual participant values. Blue indicates children with PAE and red indicates nonexposed individuals (No PAE). PAE = 27; No PAE = 10.

**FIGURE 2:**
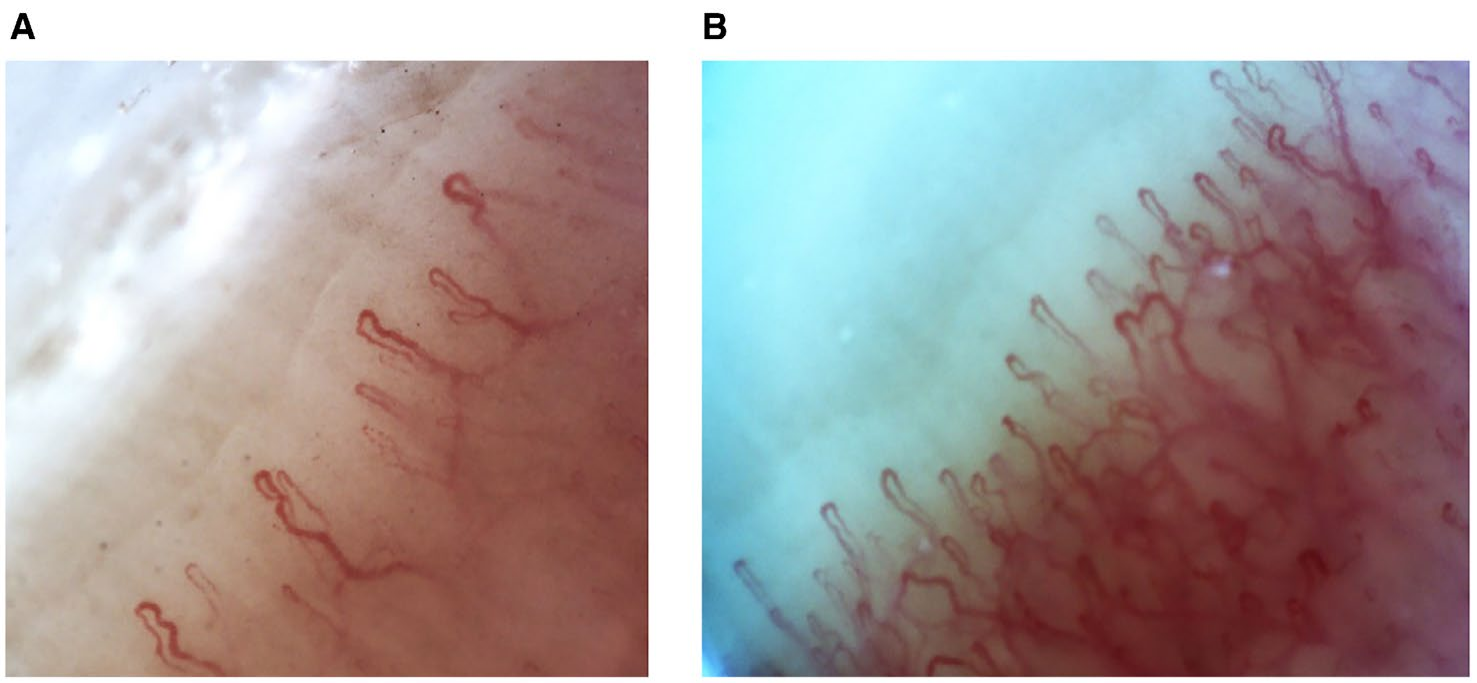
Representative nailfold capillary images from a child with prenatal alcohol exposure (A) and a nonprenatally exposed child (B), aged 3–17 years, are provided for illustrative purposes. These images demonstrate differences in capillary density observed in individual cases within the sample and are not intended to represent all participants, diagnostic findings, or population‐level patterns. All images were acquired using the same imaging settings at a standardized 200× magnification (2000 × 1500‐pixel resolution).

Table 3 shows adjusted linear regression coefficients (*β*) and 95% CI for the capillary outcomes. Figure 1 displays adjusted least‐square means and 95% CI by PAE group for capillary density (Figure 1A), intercapillary distance (Figure 1B), capillary width (Figure 1C), and enlarged capillaries (Figure 1D). After adjustment for age and sex, children with PAE demonstrated lower capillary density (see Figure 2A,B for representative nailfold capillary images) and greater inter‐capillary distance, capillary width, and percentage of enlarged capillaries compared to those without PAE. Although capillary width demonstrated a right‐skewed distribution, model residuals were acceptable. One influential observation was identified (Cook's D > 1); however, sensitivity analyses excluding this observation yielded similar findings (*β* = −8.06, *p* < 0.01), and the observation was retained in final analyses.

**TABLE 3:** Adjusted linear regression associations between prenatal alcohol exposure (PAE) and nailfold microvascular characteristics (*n* = 37).

	*β* (95% CI)	*R* ^2^	*p* value
Capillary density (capillaries/mm)	−4.43 (−6.46, −2.40)	0.40	< 0.001
Average inter‐capillary distance (μm)	38.18 (16.01, 60.36)	0.31	< 0.01
Average capillary width (μm)	9.87 (2.24, 17.51)	0.29	< 0.01
% Enlarged capillaries	28.17 (14.09, 42.25)	0.34	< 0.001

*Note:* Models adjusted for child's age and sex.

Abbreviations: *β*, adjusted linear regression coefficient; CI, confidence interval.

Age was negatively associated with capillary density (*β* = −0.22, 95% CI: −0.43, −0.01, *p* < 0.05), independent from sex and PAE group, whereas sex was independently associated with capillary width, with females demonstrating wider capillaries than males (*β* = 8.68, 95% CI: 2.05, 15.31, *p* < 0.01).

No statistically significant differences were observed for broken, branched, crossed, or multi‐crossed capillary outcomes after adjustment.

## 4 Discussion

To our knowledge, this is the first study to characterize nailfold microvascular morphology in children and adolescents with PAE. In this preliminary sample, children and adolescents with PAE exhibited a coherent and statistically significant pattern of microvascular alteration across four distinct capillary parameters: lower capillary density, wider capillary loops, greater inter‐capillary spacing, and a higher proportion of enlarged capillaries compared to unexposed peers. These findings were independent of child age and sex, and the magnitude of the effects was clinically meaningful in the context of the pediatric nailfold capillaroscopy literature.

Microvascular findings observed in the PAE group closely mirrored the signals associated with early cardiometabolic and endothelial dysfunction in other clinical populations. Microvascular rarefaction, the loss of functional capillaries, has been associated with prehypertension, Type 2 diabetes, and metabolic syndrome and is thought to both reflect and contribute to impaired tissue perfusion and endothelial dysfunction (Maldonado et al. 2017; Maranhão et al. 2016). That we observed lower capillary density in children with PAE early in life is consistent with the DOHaD hypothesis (Barker 2001, 2003), which proposes that adverse intrauterine exposures can program lasting physiological changes that increase vulnerability to cardiometabolic disease (Cook et al. 2019; Hanson and Gluckman 2008). This is supported by the growing clinical evidence base that identified a significant association between FASD and hypertension in youth independent of obesity (Cook et al. 2019; Weeks et al. 2020) and demonstrated elevated metabolic disease burden in adults with FASD; a 2026 retrospective study found significantly higher rates of structural heart remodeling, myocardial infarction, and stroke in adults with FASD relative to matched controls (Weeks et al. 2026). Our findings extend this literature by providing the first direct evidence that the peripheral microvasculature, a potential sensitive early indicator of systemic vascular health, was structurally altered in children with PAE before any overt cardiovascular diagnosis.

The density finding warrants particular attention. The PAE group was on average 3 years younger than the unexposed group, and in healthy children, capillary density decreases with age (Melsens et al. 2023; Nakajima et al. 2022). Under normal developmental conditions, the younger PAE participants would therefore be expected to exhibit *higher* density than the older unexposed peers, yet the PAE group still showed significantly lower density. Consistent with this, age was independently and negatively associated with capillary density within our full sample, consistent with some prior literature demonstrating decreases in capillary density with age (Herrick et al. 2021). This means our analysis adjusted conservatively for age, and the true effect of PAE on capillary density is likely even larger than the *β* of −4.43 capillaries/mm reported here. This conservative estimate, combined with a model R^2^ of 0.40, underscores the strength of the PAE–density association even in this small preliminary sample. Moreover, average intercapillary distance was significantly greater in those with PAE, reflecting greater spacing between adjacent capillary loops consistent with the observed reduction in density.

Enlarged capillaries are associated with inflammatory processes, autoimmune activation (e.g., rheumatic disorders), and endothelial stress in both adult and pediatric populations (Li et al. 2022; Smith et al. 2020). A meta‐analysis of NFC in pediatric autoimmune disease found that children with autoimmune conditions had nearly 28 times greater odds of dilated capillaries compared to healthy controls (Li et al. 2022). In our present study, over 70% of the PAE group's capillaries were classified as enlarged (≈30% more than the unexposed peers), and the PAE group's mean capillary width (43.9 μm) approached the 50 μm megacapillary threshold used in adult rheumatology (Komai et al. 2024; Smith et al. 2020). Whether this degree of capillary widening in children with PAE reflects ongoing low‐grade inflammation, altered angiogenesis secondary to alcohol‐induced endothelial injury during fetal development, or hemodynamic dysregulation remains an important question for future mechanistic investigation (Kable et al. 2023; Momin et al. 2023; Miranda et al. 2013; Saha and Mayhan 2022). The finding that females exhibited wider capillaries than males, independent of age and PAE group, suggests that sex‐related differences in capillary morphology may be present in this sample. Although sex was not associated with the remaining capillary measures, this finding reinforces the importance of considering sex as a covariate in NFC research, consistent with prior literature (Nakajima et al. 2022; Terreri et al. 1999).

From a clinical and translational perspective, the potential message of these findings is straightforward: children as young as 3 years old with confirmed PAE may already show a quantifiable microvascular signal that, in other at‐risk populations, is associated with future cardiometabolic morbidity. The NFC procedure is noninvasive and well tolerated by young children and is relatively inexpensive and portable. If replicated in larger samples and validated against concurrent cardiometabolic biomarkers, including blood pressure, lipids, and BMI, these capillary parameters could serve as early biosignatures for children with PAE at heightened risk for future cardiovascular or metabolic disease. This would enable earlier preventive monitoring and targeted intervention in a population that currently lacks evidence‐based early cardiometabolic screening strategies. Furthermore, given that childhood cardiovascular risk factors are strong predictors of adult cardiovascular events (Jacobs et al. 2022), identifying these risks early creates an actionable window for prevention and mitigation of future long‐term health conditions.

Several limitations must be considered. The sample is small (*N* = 37) and preliminary, with imbalance between PAE and no PAE groups (27 vs. 10). This may limit the precision of effect estimates; the present findings should be considered exploratory rather than definitive. Data are drawn from a single site with limited diversity of the sample, restraining generalizability. The observed microvascular differences may reflect unmeasured confounders or mediators, including genetic factors, postnatal environmental exposures, ambient fingertip temperature at assessment, BMI, nutritional status, physical activity, and sleep quality, all of which are known to influence NFC parameters (Jacobsen et al. 2022; Nakajima et al. 2022). The heterogeneity of PAE diagnoses within the sample may obscure dose–response or diagnosis‐specific effects. Additionally, participants without PAE were slightly older on average than those with PAE; while we adjusted for age in regression models, residual age effects cannot be entirely ruled out. Finally, established pediatric normative reference values for NFC are lacking; available normative adult data are mostly derived from European cohorts and may not be fully applicable to the racially and ethnically diverse sample studied here (Bergkamp et al. 2023). In addition, visualization of capillaries may be more difficult in individuals with darker skin tones, which could limit its use in certain populations or affect image quality and interpretation.

Future work will expand the sample to the full multisite target (*N* ≈ 200; PAE = 120, No PAE = 80) across UCSD and Emory, with increased enrollment of participants without PAE to improve balance and statistical power. As the dataset grows, analyses will examine associations between nailfold capillary parameters and concurrent clinical and subclinical cardiometabolic indicators collected on the same visit, including blood pressure, lipid profiles, glucose, hemoglobin A1c, C‐reactive protein, growth, and sleep and physical activity metrics. These analyses may also address whether cardiometabolic and inflammatory biomarkers mediate or moderate the relationship between PAE and microvascular morphology. The larger cohort will also enable evaluation of whether age modifies the association between PAE and nailfold capillary morphology, allowing assessment of potential age‐related trajectories of microvascular changes. Finally, recent studies suggest that automated and artificial intelligence‐assisted capillaroscopy analysis may reduce observer variability and improve efficiency in capillary detection, classification, and quantitative measurement, supporting the future scalability of NFC in clinical and research settings (Berks et al., n.d.; Brzezińska et al. 2024; Herrick et al. 2021). Building on this emerging work, as a next step, we are exploring the potential application of machine learning‐based image analysis to further support the use of NFC as a scalable biomarker tool in FASD settings.

## 5 Conclusion

In this first characterization of nailfold microvascular morphology in children with PAE, this exposure was associated with a coherent pattern of capillary alterations, lower density, greater inter‐capillary distance, wider capillaries, and a higher proportion of enlarged capillaries, detectable in children as young as 3 years of age, independent of age and sex. These findings suggest that subclinical microvascular compromises may be an early and measurable consequence of PAE that predicts future risk, consistent with the broader cardiometabolic risk trajectory documented in this population. Replication in larger, multisite samples and integration with concurrent cardiometabolic biomarkers are essential next steps in establishing NFC as a clinically meaningful early biomarker of cardiovascular and metabolic risk in children with PAE.

## Funding

The study is conducted under the support of the Collaborative Initiative on Fetal Alcohol Spectrum Disorders (CIFASD) https://cifasd.org/, a National Institute on Alcohol Abuse and Alcoholism (NIAAA)‐funded research consortium dedicated to advancing knowledge of the biological and clinical consequences of PAE. Research described in this manuscript was supported by Grant No. U01AA014835 to Christina Chambers funded by the NIAAA.

## Conflicts of Interest

The authors declare no conflicts of interest.

## Data Availability

The data that support the findings of this study are available from the corresponding author upon reasonable request.
